# Inpatient Complications and Outcomes for Burn Patients Admitted with Opioid-Positive Drug Screens

**DOI:** 10.1093/jbcr/irag126

**Published:** 2026-08-04

**Authors:** Emily P Swafford, Arman J Fijany, Tyler J Murphy, Punit Vyas, Jordan T Garcia, Jessica Ballou, Robel T Beyene, Stephen Gondek, Anne Wagner, Elizabeth Dale Slater

**Affiliations:** Department of Surgery, Vanderbilt University Medical Center, Nashville, TN 37232, United States; Department of Surgery, Vanderbilt University Medical Center, Nashville, TN 37232, United States; Department of Surgery, Vanderbilt University Medical Center, Nashville, TN 37232, United States; Department of Surgery, Vanderbilt University Medical Center, Nashville, TN 37232, United States; Department of Surgery, Vanderbilt University Medical Center, Nashville, TN 37232, United States; Department of Surgery, Vanderbilt University Medical Center, Nashville, TN 37232, United States; Department of Surgery, Vanderbilt University Medical Center, Nashville, TN 37232, United States; Department of Surgery, Vanderbilt University Medical Center, Nashville, TN 37232, United States; Department of Surgery, Vanderbilt University Medical Center, Nashville, TN 37232, United States; Department of Surgery, Vanderbilt University Medical Center, Nashville, TN 37232, United States

**Keywords:** burn injury, opioid use, outcomes

## Abstract

Opioid use disorder complicates the management of burn injuries, particularly in achieving adequate pain control. While prior single-institution studies have evaluated associations between opioid use and burn outcomes, multi-institutional analyses of inpatient complications remain limited. Using the American Burn Association Noncommercial Burn Research Dataset, this study compares inpatient outcomes among patients with prehospital opioid-positive screening versus those with negative screening. Among 32 052 patients included, 50.5% screened positive for opioids at the time of presentation. These patients were more likely to be female (*P* < .0001), more likely to possess scald injuries (*P* < .0001), and less likely to be experiencing homelessness (*P* < .0001) or to be diagnosed with inhalation injury (*P* < .005). Opioid positivity was associated with increased odds of infectious complications, such as cellulitis (OR = 2.7, *P* < .0001) and sepsis (OR = 1.41, *P* < .001). Opioid negativity, conversely, had a higher prevalence of acute respiratory distress syndrome (*P* < .0001), acute kidney injury (*P* < .0001), respiratory failure (*P* < .05), and cardiac arrest (*P* < .05). The opioid-negative population also necessitated more frequent admission to the critical care unit (*P* < .05) and demonstrated higher overall mortality (*P* < .0001). These findings highlight the vulnerability of opioid-negative patients who may have delayed presentation and more severe injuries than originally anticipated. This work underscores the need for heightened vigilance and equitable, early care for these patients to mitigate risk in this group.

## INTRODUCTION

Burn injuries are among the most severe in terms of morbidity and mortality, causing approximately 180 000 deaths annually.^1^ The pain associated with burns is recognized as one of the most intense and challenging to manage given its complex, multifactorial nature.^2–4^ Burn injuries result in both nociceptive and neuropathic pain, and their treatment often involves painful interventions.^5^ Poor pain management delays healing by triggering cortisol and catecholamine release, resulting in prolonged hospital stays.^5^^,^^6^ Opioids remain first-line in the management of burn pain, with a multimodal approach being the most effective.^4^^,^^7^

Despite growing awareness, opioid use disorder remains widespread in the United States. In 2023, the Centers for Disease Control and Prevention reported that 8.6 million Americans aged 12 years and older misused prescription opioids.^8^ Opioid overdose-related deaths surpassed 100 000 annually.^9^ Drug intoxication and chronic opioid use can complicate the management of patients with burn injuries due to challenges in managing severe pain. For burn surgeons, balancing effective pain management while minimizing opioid-related side effects, such as respiratory depression, remains a critical challenge, especially in patients who are not opioid naive.

While prior studies have explored the effects of opioid use on burn outcomes, there has yet to be a multi-institutional database study focused on inpatient complications. This study aims to compare inpatient outcomes in patients with prehospital opioid-positive screening to those with negative screening.

## METHODS

This study analyzed the American Burn Association Noncommercial Burn Research Dataset for burn admissions with opioid-positive drug screens from 2012 through 2021. Patient demographics, injury characteristics, complications, and outcomes were extracted. Patients who did not receive opioid screening were excluded. *Opioid positivity* was defined as a positive result on opioid drug screen at time of presentation.

The primary outcome of interest was mortality, while secondary outcomes included cellulitis, pneumonia, systemic sepsis, acute respiratory distress syndrome (ARDS), respiratory failure, unplanned intubation, acute kidney injury (AKI), renal failure, alcohol withdrawal, arrhythmia, cardiac arrest, deep vein thrombosis, and intensive care unit (ICU) admission. Univariable analysis identified potential factors associated with opioid positivity that were included in subsequent subgroup analyses to assess for significant association. Analysis with multivariate logistical regression was used to describe associations between opioid positivity and primary and secondary outcomes. Covariate adjustments were made for primary and secondary outcomes and included age, sex, percentage of total body surface area (TBSA) burned, and inhalation injury. For primary and secondary outcomes included in covariate analyses, patient admissions that lacked data for any of the four covariates were dropped from the analysis.

All primary and secondary outcome results derived from multivariate regression were reported with odds ratios (ORs) with 95% confidence intervals. Chi-square test was used to calculate OR. All statistical analysis was performed in PyCharm 3.1 software using pandas, NumPy, and SciPy. Stats modules with significance set as *P* < .05.

## RESULTS

Between 2012 and 2021, 285 544 patients were included in the dataset. Thirty-two thousand and fifty-five patients possessed drug screen data. Of those, 16 201 (50.5%) were found to be opioid positive at their time of arrival. Of patients who screened positive, 71.3% were male with a mean age of 42 years old. 10 275 (63.4%) identified as White, 3394 (20.9%) as Black, 199 (<2%) as American Indian/Alaskan/Native or Asian, and 1490 (9.2%) as Other. 2.8% of patients denied stable housing (Figure 1).

**Figure 1:**
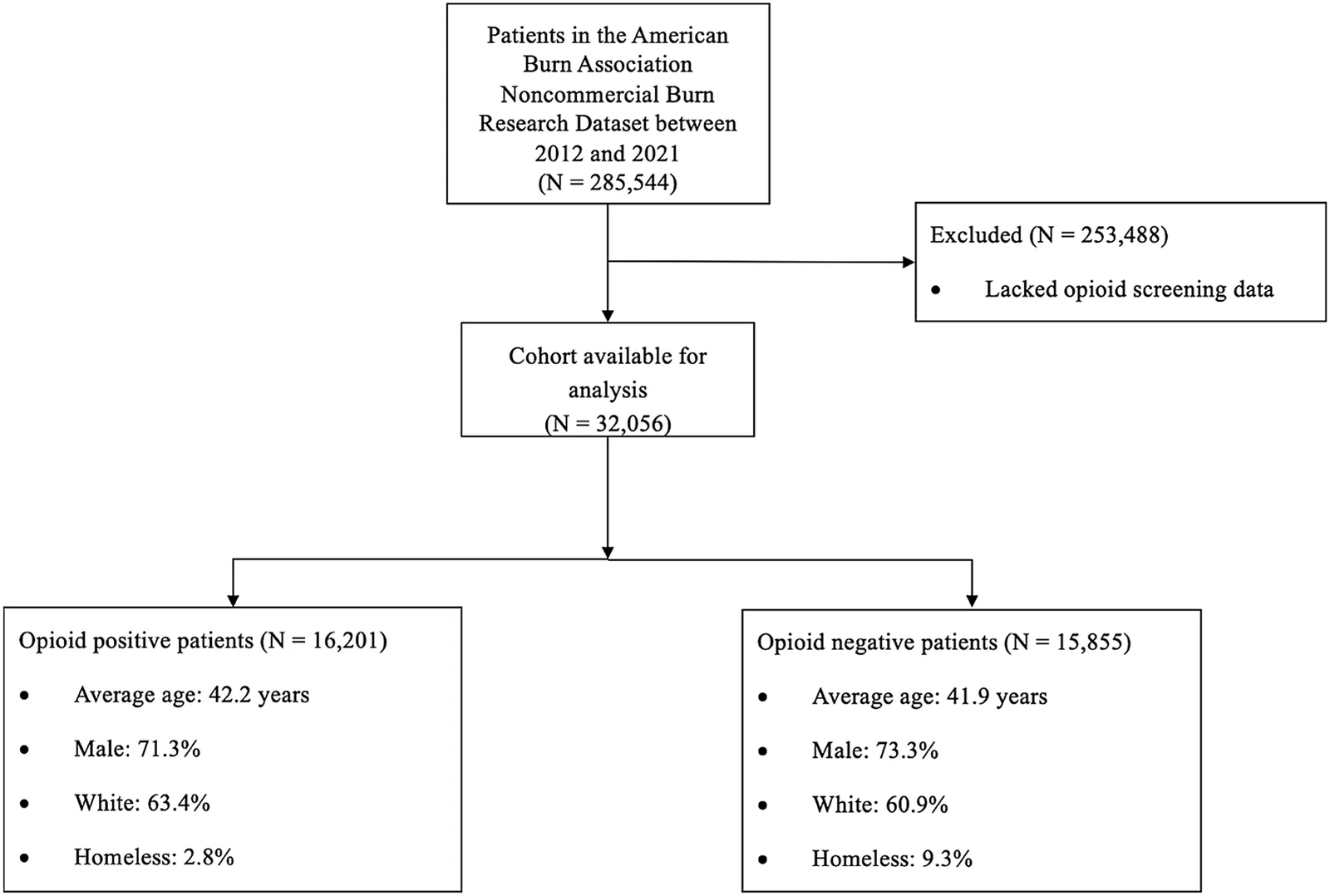
Patient Flow Diagram

Univariate analysis revealed that opioid-positive patients were more likely to be white (*P* < .0001) and female (*P* < .0005). They were also more likely to have a scald burn (*P* < .001), chemical burn (*P* < .001), or contact injury (*P* < .001). Patients who lacked opioid positivity were more likely to be experiencing homelessness (*P* < .0001), more likely to have flame burns (*P* < .0001), and more likely to have inhalation injury (*P* < .005). Opioid positivity was not significantly associated with age, electrical burns, or cold injury (Table 1).

**Table 1:** Demographic and Clinical Characteristics of the Study Population

	**Opioid positive (*N* = 16 201)**	**Opioid negative (*N* = 15 855)**	***P* value**
Age (years)	42.2 (±16.7)	41.9 (±15.8)	.15
Male	11 554 (71.3)	11 625 (73.3)	**<.0005**
Homeless	461 (2.8)	1469 (9.3)	**<.0001**
Race			
White	10 275 (63.4)	9650 (60.9)	**<.0001**
Black	3394 (20.9)	3257 (20.5)	.38
American Indian/Alaskan/Native	311 (1.9)	176 (1.1)	**<.0001**
Asian	169 (1.0)	159 (1.0)	.76
Native Hawaiian/Pacific Islander	30 (0.2)	49 (0.3)	**<.05**
Other	1490 (9.2)	1474 (9.3)	.77
Inhalation injury	27 (0.2)	57 (0.4)	**<.005**
Flame burn	6801 (42.0%)	7705 (48.6%)	**<.0001**
Scald burn	5072 (14.5)	3661 (22.6)	**<.001**
Electrical burn	989 (2.8)	451 (2.8)	.555
Chemical burn	826 (2.4)	415 (2.6)	**<.001**
Cold injury	600 (1.7)	190 (1.2)	.991
Contact injury	2019 (5.8)	1012 (6.2)	**<.001**

Values expressed as *N* (%) or Mean (SD) unless otherwise indicated. Bold represents significant values.

Patients who tested positive for opioids at the time of arrival were associated with increased odds of developing cellulitis (*P* < .0001) and sepsis (*P* < .0001). However, those who did not test positive were associated with increased risk of developing ARDS (*P* < .0001), AKI (*P* < .0001), and cardiac arrest (*P* < .05) (Table 2). After adjusting for critical confounding factors such as age, sex, percent TBSA, and inhalation injuries, opioid-negative patients were also more likely to experience respiratory failure (*P* < .05) and require an ICU admission (*P* < .0001). They had a statistically significant increase in mortality rate (*P* < .0001) when compared to opioid-positive patients (Table 3).

**Table 2:** Associated Complications of Opioid Positivity

**Complication**	**Opioid positive**	**Not opioid positive**	**Odds ratio**	***P* value**
Cellulitis	239 (3.8)	117 (1.5)	2.7	<.0001
Pneumonia	624 (10.0)	820 (10.2)	0.98	.72
Systemic sepsis	360 (5.8)	335 (4.2)	1.41	<.0001
ARDS	114 (1.8)	233 (2.9)	0.62	<.0001
Unplanned intubation	90 (1.4)	139 (1.7)	0.83	.20
AKI	1 (0.0)	98 (1.2)	0.01	<.0001
Renal failure	223 (3.6)	253 (3.2)	1.14	.17
Alcohol withdrawal	74 (1.2)	76 (0.9)	1.26	.19
Arrhythmia	136 (2.2)	189 (2.4)	0.93	.53
Cardiac arrest	121 (1.9)	205 (2.6)	0.76	<.05

Abbreviations: AKI, acute kidney injury; ARDS, acute respiratory distress syndrome.

**Table 3:** Multivariate regression outcomes

	**Opioid negative**	**Opioid positive**	***P* value**
Mortality	744 (5.5)	454 (3.2)	<.0001
DVT	204 (2.8)	135 (2.4)	.21
Respiratory failure	133 (1.8)	127 (2.3)	<.05
ICU	7369 (75.4)	5383 (59.3)	<.0001

Abbreviation: DVT, deep vein thrombosis.

## DISCUSSION

This study provides a comprehensive, multi-institutional analysis of inpatient outcomes among burn patients who screened for opioids at presentation. Our findings demonstrate that opioid-negative patients experience higher rates of severe complications, including ARDS, AKI, respiratory failure, and cardiac arrest. These patients also require more frequent ICU admission and possess a higher overall mortality rate, consistent with a more critically ill cohort.

These results challenge the assumption that opioid positivity on initial screening reflects chronic use or opioid tolerance. A positive drug screen is a limited, binary measure that cannot distinguish between acute, prehospital administration, or established, long-term use. Instead, opioid positivity may function as a proxy for earlier access to medical care. This interpretation is supported by our finding that patients with more overt injuries—such as scald or chemical burns—were more likely to receive opioids prior to arrival compared with those with less externally apparent but potentially more severe injuries, such as inhalation injury.

The observed differences may also reflect broader structural disparities as opioid-negative patients were more likely to experience homelessness. Prior work has demonstrated that individuals with housing instability and lower socioeconomic status have reduced access to emergency medical services.^10–12^ These disparities are further compounded by well-described bias in pain assessment and management in this population. Despite a higher burden of baseline illness and chronic pain, patients experiencing homelessness are frequently undertreated due to concerns surrounding drug-seeking behavior or medication misuse, resulting in additional barriers to adequate analgesia.^13^^,^^14^ Moreover, substandard housing conditions may increase both the incidence and severity of burn injury. The lack of reliable heating sources or functional fire alarms may necessitate unsafe alternatives, such as space heaters, and prevent awareness of a fire, leading to delay extrication and contributing to flame burns and inhalation injuries—both of which are more commonly observed in the opioid-negative cohort.^10–12^ In this context, the absence of prehospital opioid exposure may more accurately reflect limited access to care rather than differences in substance use.

A major strength of this study is its use of a large, multi-institutional cohort, which allows for comprehensive assessment of the association between opioid screening status and outcomes across a diverse patient population. The large sample size provides robust statistical power capable of detecting clinically meaningful differences in mortality and major inpatient complications. Meanwhile, the inclusion of patients from multiple burn centers enhances the external validity and generalizability of the findings. Additionally, this work evaluates multiple outcomes—including organ dysfunction, respiratory complications, ICU utilization, and mortality—offering a more complete assessment of the clinical impact of opioid exposure in burn patients as opposed to studies with a single endpoint.

Nevertheless, several limitations should be considered when interpreting these findings. Firstly, the dataset could not distinguish among sources of opioid exposure, including prehospital or transfer-related opioid administration, medication-assisted treatment for opioid use disorder (eg, methadone or buprenorphine therapy), prescribed opioid use, and nonmedical opioid use. Consequently, opioid positivity likely represents a heterogeneous population, introducing the potential for exposure misclassification. Secondly, only 11% of the patients in dataset possessed opioid screening data, raising the possibility of selection bias if the patients chosen for screening were systematically different from those who were not tested. Finally, although opioid exposure was the primary variable of interest, concurrent use of other substances—including cocaine, methamphetamine, cannabis, benzodiazepines, and other illicit or prescribed agents—may independently influence injury patterns, physiologic response, and clinical outcomes. The inability to fully account for polysubstance use may therefore have contributed to residual confounding.

Overall, these findings highlight the vulnerability of opioid-negative patients who may have delayed presentation with more severity than originally anticipated. This work underscores the need for heightened vigilance and equitable, early care for patients with less visible but high-risk injuries. Future research should aim to clarify the nuanced relationship between opioid use and burn outcomes by distinguishing the source of opioid administration in positive patients, leveraging longitudinal datasets, and exploring methods to improve outcomes in this population.

## CONCLUSION

To our knowledge, this is the largest multi-institutional analysis exploring the effects of prehospital opioid use on patient outcomes for burn patients. The higher prevalence of ARDS, AKI, respiratory failure, cardiac arrest, and overall increased rate of mortality in the opioid-negative patient population highlights the vulnerability of this group to complications. Increased awareness of these complications may mitigate risk in this group.

## Notes

Author contributions: Emily P. Swafford (Conceptualization [equal], Data curation [equal], Formal analysis [equal], Writing—original draft [lead]), Arman J. Fijany (Conceptualization [equal], Data curation [lead], Formal analysis [lead], Writing—review & editing [equal]), Tyler J. Murphy (Conceptualization [equal], Data curation [equal], Formal analysis [equal]), Punit Vyas (Conceptualization [equal], Data curation [equal], Formal analysis [equal]), Jordan T. Garcia (Conceptualization [equal], Data curation [equal], Formal analysis [equal]), Jessica H. Ballou (Conceptualization [equal], Writing—review & editing [equal]), Robel T. Beyene (Conceptualization [equal], Data curation [equal], Formal analysis [equal], Writing—review & editing [equal]), Stephen P. Gondek (Conceptualization [equal], Data curation [equal], Formal analysis [supporting], Writing—review & editing [equal]), Anne Wagner (Conceptualization [equal], Data curation [equal], Formal analysis [equal], Writing—review & editing [equal]), Elizabeth Dale Slater (Conceptualization [equal], Data curation [equal], Formal analysis [equal], Writing—review & editing [equal])

Funding: *None declared.*
